# Two-faced Janus: the dual role of macrophages in atherosclerotic calcification^[Author-notes cvab301-FM2]^

**DOI:** 10.1093/cvr/cvab301

**Published:** 2021-09-22

**Authors:** Olivia J Waring, Nikolaos T Skenteris, Erik A L Biessen, Marjo M P C Donners

**Affiliations:** Department of Pathology, Cardiovascular Research Institute Maastricht (CARIM), Maastricht University Medical Center, P. Debyelaan 25, 6229 HX, Maastricht, The Netherlands; Department of Pathology, Cardiovascular Research Institute Maastricht (CARIM), Maastricht University Medical Center, P. Debyelaan 25, 6229 HX, Maastricht, The Netherlands; Cardiovascular Medicine Unit, Department of Medicine, Karolinska Institutet, Visionsgatan 4, 171 64, Solna, Sweden; Department of Pathology, Cardiovascular Research Institute Maastricht (CARIM), Maastricht University Medical Center, P. Debyelaan 25, 6229 HX, Maastricht, The Netherlands; Institute for Molecular Cardiovascular Research, RWTH Aachen University, Templergraben 55, 52062, Aachen, Germany; Department of Pathology, Cardiovascular Research Institute Maastricht (CARIM), Maastricht University Medical Center, P. Debyelaan 25, 6229 HX, Maastricht, The Netherlands

**Keywords:** Macrophages, Inflammation, Calcification, Vascular remodelling, Atherosclerosis

## Abstract

Calcification is an independent predictor of atherosclerosis-related cardiovascular events. Microcalcification is linked to inflamed, unstable lesions, in comparison to the fibrotic stable plaque phenotype generally associated with advanced calcification. This paradox relates to recognition that calcification presents in a wide spectrum of manifestations that differentially impact plaque’s fate. Macrophages, the main inflammatory cells in atherosclerotic plaque, have a multifaceted role in disease progression. They crucially control the mineralization process, from microcalcification to the osteoid metaplasia of bone-like tissue. It is a bilateral interaction that weighs heavily on the overall plaque fate but remains rather unexplored. This review highlights current knowledge about macrophage phenotypic changes in relation to and interaction with the calcifying environment. On the one hand, macrophage-led inflammation kickstarts microcalcification through a multitude of interlinked mechanisms, which in turn stimulates phenotypic changes in vascular cell types to drive microcalcification. Macrophages may also modulate the expression/activity of calcification inhibitors and inducers, or eliminate hydroxyapatite nucleation points. Contrarily, direct exposure of macrophages to an early calcifying milieu impacts macrophage phenotype, with repercussions for plaque progression and/or stability. Macrophages surrounding macrocalcification deposits show a more reparative phenotype, modulating extracellular matrix, and expressing osteoclast genes. This phenotypic shift favours gradual displacement of the pro-inflammatory hubs; the lipid necrotic core, by macrocalcification. Parallels to bone metabolism may explain many of these changes to macrophage phenotype, with advanced calcification able to show homeostatic osteoid metaplasia. As the targeted treatment of vascular calcification developing in atherosclerosis is thus far severely lacking, it is crucial to better understand its mechanisms of development.

## 1. Introduction

Atherosclerosis is a slowly progressing, chronic inflammatory disease that affects large- and middle-size arteries,^1^ featuring the accumulation of fatty and fibrous elements together with immune cells, and structural vascular smooth muscle cells (VSMCs) in the intimal layer of the arterial wall. During disease progression, atherosclerotic plaques develop regions of mineralization, a process which has been traditionally linked to an increased risk for heart disease, atherosclerotic plaque rupture, and stroke.^2,3^ Rather than a mere by-product of the development and changing inflammatory environment of the plaque, calcification impacts grievously on disease progression and pathogenesis, particularly through mediating biomechanical destabilization and directly impacting plaque inflammation. Calcification, be it bone related or ectopic, is an active process involving interplay between multiple cell types,^4^ with an important role for osteoclast-like macrophages in bone. Osteoclasts, the specialized resorptive cells found in bone, derived from a common myeloid progenitor with macrophages. In plaque, both multinucleated giant cells and macrophages are observed to emulate osteoclast traits^5–9^ induced through the RANK/RANK-L/OPG signalling axis,^10,11^ as present in late-stage calcification.

Although micro- and macrocalcification often occur side-by-side during plaque progression, microcalcification is largely observed in earlier-stage lesions,^12^ while the latter predominates in late-stage plaque.^13^ Microcalcification particles, defined as <50 μm in size,^14^ are developed in a four-stage process, involving calcifying extracellular vesicle (cEV) accumulation, aggregation, membrane fusion, and finally, mineralization.^15^ During mineralization, amorphous calcium phosphate transforms into mature crystal-like form hydroxyapatite ‘microcalcification’ particles, present in spherical and needle-like morphology types, 0.5–15 µm in size.^16^ They become larger as lesions progress, as ‘speckled calcification’ (≥15 µm to ≤2 mm in diameter).^17^ Microcalcification particles in the fibrous cap increase the risk of plaque rupture.^14,18^ These particles coalesce into larger sheet-like or nodular structures, up to several millimetres in diameter. Such macrocalcification has been linked with healing response and plaque stability.^19,20^ However, increased coronary artery calcification (CAC) score is related to atherosclerotic plaque burden, has been linked with all-cause mortality and is a broadly adopted predictor of cardiovascular events.^21,22^

Like Janus, the Roman God of duality, macrophages in the atherosclerotic plaque are seen to have both accelerative and decelerative, bilateral relationship with calcification. On the one hand, they may trigger and exacerbate vascular calcification onset, as calcification first develops in inflammatory hotspots throughout the plaque^23^; whereas on the other hand, macrophages may limit calcification by encapsulation, internalization, and resorption of macro^24^ and microdeposits.^25^

Macrophages co-localize with calcium phosphate crystals in developing atherosclerotic lesions.^26–28^ The presence of inflammatory macrophages has even been used as a surrogate marker for early microcalcification.^29^ Vice versa, a high score of intimal microcalcification can help to pinpoint the most inflamed^28^ and likely to rupture plaque areas.^30^ Multimodal ^18^F-NaF and ^18^F-Fluorodeoxyglucose PET imaging of both measures allows detection of highly metabolically active inflamed areas and microcalcified areas in plaque in one shot.^31^ In contrast, areas of macrocalcification have been largely observed to feature fewer inflammatory cells, more reparative macrophages, including osteoclast-like cells,^6^ more fibrosis,^32^ and presentation of osteoid metaplasia.^33^ This was confirmed by transcriptional analysis of human high- vs. low-calcified carotid atherosclerotic plaques, showing repressed inflammation, lipid transport, and chemokine signalling pathways.^13^ Hence, a better comprehension of exactly how macrophages engage with calcification throughout disease progression will offer more opportunities for highly necessary, novel therapeutics. In this review, we will outline current literature on macrophage crosstalk with intimal calcification in atherosclerosis, including both direct and indirect interactions, and its impact on disease progression.

## 2. Macrophage phenotypic plasticity in response to a calcified microenvironment

Macrophages’ remarkable plasticity and functional heterogeneity render them adaptive, according to specific microenvironment stimuli, to different subsets or phenotypes.^34^ To understand how macrophages behave in calcified plaques, *in vitro* assessment of macrophage response to individual calcifying stimuli has been performed. In this, it is important to note that the outcome appears to greatly depend on the initial phenotype of the cells being studied, but this is not regularly factored into account. The M1/M2 macrophage model,^35^ whilst being now considered a too-broad descriptor of macrophage’s full phenotypic spectrum, has been shown to have opposing effects on extracellular endogenous mechanisms of calcification as further elaborated on below and may respond differently to a calcified/calcifying environment.

Attempting to mimic macrophage responses to a microcalcified environment, some *in vitro* studies have shown M2-like phenotypic shift in hyperphosphataemia. Macrophages had increased phosphate-handling ability and enhanced arginine hydrolysis, which both may dampen crystal nucleation within the plaque.^36^ These phosphate-polarized cells produced higher levels of secreted adenosine triphosphate (ATP) and increased pyrophosphate (PPi) synthesis, inhibiting calcium phosphate deposition.^37^ PPi is produced by the enzyme ectonucleotide pyrophosphatase/phosphodiesterase 1 (eNPP), which hydrolyses extracellular ATP to generate PPi and adenosine monophosphate. Therefore, PPi inhibits the precipitation of calcium phosphate, preventing the formation of hydroxyapatite and favouring its dissolution. Phosphate-polarized cells also showed enrichment in oxidative stress handling genes.^36^

Inversive to the response seen to hyperphosphataemia, incubation of macrophages with calcium phosphate-supplemented medium could induce the release of calcifying matrix extracellular vesicles, and increased interleukin (IL)-6 expression in M1 polarized cells, while M2 polarized cells had reduced induction of Arginase-1 expression upon the same stimulation,^38^ both pointing to net M1 skewing. Alone, increased extracellular Ca^2+^ could trigger NLR family pyrin domain containing 3 (NLRP3) activation in monocytes and increase IL-1β secretion after lipopolysaccharide stimulation.^39^ However, the combined exposure to the pro-inflammatory cytokine tumour necrosis factor-alpha (TNF-α), plus CaPO_4_ stimulated the transformation of macrophages into osteoclast-like cells *in vitro*, in an RANK-L independent manner.^40^ Importantly*, in vitro* cell culture models reliant on supplementing additional calcium phosphate to alter normal equilibrium may better reflect a medial calcification environment, as it is observed during kidney dysfunction.^41^

Calcium phosphate crystals can be internalized actively by human monocyte-derived macrophages through phagocytosis, induce a pro-inflammatory M1 phenotype, and activate the NLRP3 inflammasome complex to release IL-1β, amongst others.^25,42,43^ IL-1 molecule release in response to cholesterol crystal phagocytosis and NLRP3 activation drives the recruitment of neutrophils, and early lesion formation.^44^ This pro-inflammatory response to calcium phosphate particles could be reversed/dampened by co-incubation with Fetuin A or Gla-Rich Protein (GRP), both natural calcification inhibitors.^45,46^ Stimulation of THP-1 derived macrophages with hydroxyapatite nanoparticles, the naturally occurring mineral form of calcium phosphate, alone could also induce the expression of GRP and Matrix Gla Protein (MGP),^46^ a potent vitamin K-dependent protein inhibitor of vascular calcification produced by VSMCs and chondrocytes. The mechanism behind the hydroxyapatite induced pro-inflammatory response is not fully understood, nor is it known if macrophages can directly sense and respond to hydroxyapatite particles, or if pro-inflammatory responses are instead a by-product of frustrated phagocytosis due to the inability to effectively breakdown ingested hydroxyapatite particles. However, the physicochemical properties of hydroxyapatite particles are highly variable, with sizes ranging from 0.1 to 100 µm and needle-shaped/spherical morphology and smooth/rough surface topology, all factors that can modulate the degree of inflammatory response.^47^

As the atherosclerotic plaque milieu is so complex and difficult to model in culture, it is still largely unclear how direct macrophage-calcification stimulation seen *in vitro* is taking place in the inflammatory state of the plaque itself. Particularly, as plaque macrophages are likely to be highly inflammatory, this may skew responses to calcification stimuli *in vivo*. Deep phenotyping studies of plaque macrophages in proximity to early and advanced plaque calcification would help mapping this causality dilemma. Several ground-breaking single-cell sequencing studies in atherosclerotic plaque have helped to highlight the macrophage spectrum in this disease,^48–53^ but as of yet, studies comparing cellular heterogeneity of calcified vs. non-calcified plaques are lacking. A second outstanding question is to what extent the calcification-related macrophage phenotype *in vivo* is dependent on the physicochemical features of the calcium phosphate particle (e.g. charge, size, composition). The distinct response of macrophages to hydroxyapatite particles and inorganic minerals suggests that pathological atherosclerotic calcification is not merely a passive consequence of chronic inflammatory disease but may lead to a positive feedback loop as a result of the active interplay between calcification and inflammation during the disease progression.

## 3. Macrophage contribution to intimal calcification

The critical step in the formation of an atherosclerotic plaque is the infiltration of macrophages in the subendothelial space. In this sense, macrophage infiltration is a sine qua non for vascular calcification. Direct causal involvement of macrophages in vascular calcification is conceivable. This section will review the diverse mechanisms in microcalcification initiation (i) cEV release, (ii) apoptotic body nucleation, (iii) endogenous inhibitor dysregulation, and (iv) osteogenic transdifferentiation, involving both direct and indirect macrophage engagement. All these mechanisms, occurring simultaneously in actively calcifying plaques, have been shown to be initiated and driven by macrophage interaction with the microenvironment and contained cells.

### 3.1 Macrophage extracellular vesicles

Macrophages can directly contribute to atherosclerotic plaque calcification through the release of cEVs.^38,54^ These macrophage extracellular vesicles are characterized by markers CD9, CD63, CD81, TSG101, and CD68,^38,55^ externalized phosphatidylserine, and are loaded with S100A9 and Annexin-5 proteins. As well as possessing high calcification potential, accumulation and aggregation of cEVs initiate nucleation of hydroxyapatite particles, promoting the mineralization process within plaques.^56^

Parallels between extracellular vesicles released in the pro-inflammatory atherosclerotic plaque milieu, and matrix vesicles in bone formation can be drawn, since they share many commonalities; high mineralization potential, annexin expression, and acidic lipids such as phosphatidylserine.^57,58^ However, extracellular vesicles are highly variable; recent high-throughput technologies highlighted phenotypic differences, consistent with their originating cell type; including immunopositivity for cell markers, protein, and RNA content.^59^ Furthermore, differences between vesicles from the same origin cells can be seen in an altered microenvironment. Comparative proteomic profiling analysis of extracellular vesicles released from primary mouse aortic smooth muscle cells upon different pro-osteogenic conditions demonstrated significant differences in protein composition, such as endocytosis-associated proteins reduced vesicles released from phosphate-stimulated cells.^60^ In agreement, proteomic analysis of cEVs from human VSMCs and valvular interstitial cells cultured in osteogenic media revealed an enrichment of annexins including ANXA1 and its calcium-dependent binding partner, S100 calcium-binding protein A11 (S100A11) that could tether extracellular vesicles.^61^ Interestingly, ANXA1 knockdown attenuated extracellular vesicle microcalcification and therefore human SMCs and VICs calcification.^61^ More research to that direction is needed in the developing multi-omics era, for further characterization of these vesicles, their loading molecules as well as their emerging role in and beyond the vascular calcification pathology. Extracellular vesicles’ ability to contain proteins, lipids, nucleic acids, and other signalling molecules, as well as their capability to circulate and transmit specific molecular information to other cell types influencing their function, is of great interest for potential and promising diagnostic and prognostic biomarker evaluation.^62^

### 3.2 Macrophage lipid handling and cell death driving microcalcification

Lipid infiltration and modification in early atherosclerosis trigger an inflammatory response, monocyte recruitment, and macrophage differentiation, as well as foam cell generation.^63,64^ The lipid-rich necrotic core is a key site of early calcification, with high hydroxyapatite nucleation potential.^65,66^ Lipids may enhance the deposition of calcium crystals serving as an extra scaffold for calcification, by triggering osteogenic differentiation of VSMCs. Similarly, by affecting foam cell efferocytosis and stimulating inflammation, the lipid core increases the calcification propensity of surrounding cells and extracellular matrix. Apoptosis of VSMCs in culture was shown to be a key regulator of the initiation of vascular calcification with apoptotic bodies acting as nucleation sites for calcification.^67^ Parallels have also been drawn between apoptotic bodies and matrix microvesicles that induce calcification in bone.^68^ Failure of macrophages to clear apoptotic bodies, as observed in advanced atherosclerosis,^69,70^ allows calcium crystal growth to progress, and may also be a significant inflammatory spur leading to the release of cytokines such as TNF-α, also a potent inducer of osteogenic gene expression in VSMCs.^71^

Uptake of ox-LDL triggers apoptosis in macrophages and VSMCs.^72,73^ In early lesions, macrophage apoptosis can reduce overall plaque size and lesion inflammation,^74^ however, in more advanced lesions, with compromised efferocytosis, apoptosis will transition to secondary necrosis, which is detrimental to plaque development and increases calcification.^75,76^ Macrophages are highly effective in efferocytosis and have a high capacity for continued clearance of apoptotic cells in the plaque; upon uptake of apoptotic cells, they release anti-inflammatory cytokines IL-10 and transforming growth factor-beta (TGF-β).^77^ Thus, targeting efferocytosis in macrophages may have promise in reducing vascular calcification, as well as overall plaque progression. Efferocytosis-targeting strategies such as blockage of CD47 ‘don’t eat me’ signalling dramatically reduced atherosclerosis in ApoE^−/−^ through the improvement of debris clearance by macrophages.^78^ CD47 inhibition is already considered for cancer therapy, making clinical translation to atherosclerosis potentially easier^79^ and recently CD47-interference nanotherapy was shown to have a favourable outcome in atherosclerotic ApoE^−/−^ mice.^80^

Beyond influencing cell death, macrophage lipid handling also impacts the inflammatory nature of the plaque. OxLDL has a chemotactic effect on monocytes, and is a TLR4 agonist^81^; stimulation of both macrophages and VSMCs with oxLDL increases their expression of TLR4.^82,83^ It activates NF-kB signalling, producing a pro-inflammatory phenotype in macrophages, and increasing osteoblastic differentiation and calcification in VSMCs,^84^ as well as increasing foam cell formation in both. Moreover, oxLDL uptake and subsequent lysosomal cholesterol crystal generation are inflammasome activating factors in macrophages, allowing maturation and secretion of IL-1β and IL-18.^85,86^ IL-1β production is a key factor in perpetuated atherosclerotic calcification, as mentioned, as it is also induced in response to hydroxyapatite stimulation,^87^ suggestive of positive feedback during microcalcification establishment. Indeed therapeutic inhibition of IL-1β in Ldlr^−/−^ mice using a monoclonal antibody showed greatly diminished calcification burden within plaques.^88^ Inflammasome associated IL-1β production is kept in check by Rho GTPases RAC1 and 2, the expression of which was seen to be down-regulated with plaque progression, potentially accelerating atherosclerotic calcification.^87^ A neutralizing IL-1β antibody increased macrophage presence within the fibrous cap and promoted M2 macrophage polarization; whereas IL-1 signalling in VSMCs is essential for their migration and collagen secretion into the fibrous cap in advanced atherosclerotic plaques.^89^ Of interest, no difference in the lesion calcification was observed, compared to IL-1 signalling and inflammasome modulation in early plaques.^90^

### 3.3 Macrophage impact on endogenous calcification inhibitors

Several endogenous mechanisms exist throughout the body to prevent ectopic calcification. Macrophage-driven inflammation causes several vascular cell types—including smooth muscle cells, endothelial cells and pericytes—to undergo phenotypic changes resulting in altered expression of calcification modulating factors.^91–93^ Macrophage-produced inflammatory drivers initiate simultaneous loss of VSMC-expressed calcification inhibitors, such as MGP, osteopontin (OPN) and PPi, and gain of inducers such as osteoprotegerin (OPG).^2^ Stimulation of VSMCs with macrophage conditioned medium simultaneously increased bone morphogenic protein-2 (BMP-2) and inhibited MGP expression.^94^

As in macrophage response to a calcified microenvironment, polarized macrophages can exert opposing pro- and anti-calcifying activity via endogenous inhibitors. M1 macrophages have higher expression and activity of the enzyme ectonucleoside triphosphate diphosphohydrolase 1 (eNTPD1, a.k.a. CD39), which hydrolyses ATP to AMP and Pi.^95^ Thus, macrophages may promote calcification by not only producing Pi, a calcification substrate, but also lowering ATP availability for eNPP1 to produce PPi,^96^ a potent calcification inhibitor. In the aortic wall, more than 90% of extracellular ATP is degraded to Pi,^95^ at a rate 10 times more rapid than the rate of PPi synthesis and insufficient for the inhibition of hydroxyapatite formation.^97^ Co-culture of M1 macrophages, or M1-derived TNF-α enhances the TNAP activity of VSMCs; augmenting calcification *in vitro.*^98^ Contrastingly, co-culture of VSMCs with M2 macrophages stimulated the synthesis of extracellular ATP and PPi and enhanced the activity of eNPP1 in VSMCs.^37^

Macrophages secrete large amounts of the inhibitors OPN and Fetuin-A in calcified plaques, which have been suggested to enhance microcalcification opsonization for the purposes of phagocytosis.^99^ Although OPN can have pro-atherogenic effects,^100^ it has been shown to be anti-calcifying in atherosclerosis, and specifically in macrophages can induce carbonic anhydrase II expression, attenuate inflammatory activation, and regulate osteoclast formation.^101,102^ Exogenous OPN exerts a significant role as an inflammatory mediator of vascular injury; it is induced in the differentiation of peripheral monocytes into an M2-like phenotype.^102^

M2 macrophages release anti-inflammatory mediators and phagocytize necrotic fragments or apoptotic cells to prevent the formation of calcified nucleation sites.^103^ Of interest, macrophage-derived OPN binding to calcium phosphate or hydroxyapatite particles functions as an opsonin^104^ and facilitates their ingestion through the phagocytosis process. In accordance, fetuin/α2-HS glycoprotein, another vascular calcification inhibitor, enhances phagocytosis of apoptotic cells and macropinocytosis by macrophages,^105^ reducing the accumulation of pro-calcifying apoptotic vesicles.

### 3.4 Macrophage impact on smooth muscle cell osteogenic transdifferentiation

Macrophage interaction with VSMCs heavily contributes to plaque calcification and is perhaps their most impactful indirectly calcifying activity. Macrophages release a vast variety of pro-osteogenic cytokines^71,106,107^ that stimulate smooth muscle cells to transdifferentiate into an osteogenic phenotype. VSMC-osteo/chondrogenic phenotype^94^ is accompanied by genetic lineage reprogramming involving up-regulation of osteochondrogenic markers (RUNX2, SOX9 ALP, osteocalcin, osterix, type II, and X collagen), down-regulation of VSMC markers (SM22a, SMa actin, etc.),^108^ and secretion of calcifying microvesicles.^109^

It was shown that co-culture of macrophages with VSMCs profoundly affected the ability of the latter to calcify. Inflammatory macrophages especially induced VSMC chondrogenic switch, as well as active calcification.^110^ Co-culture of VSMCs with murine M2 macrophages, however, inhibited calcification.^37^ Paradoxically, M2 hallmark cytokines such as TGF-β have also been reported to have pro-calcifying effects: direct stimulation of VSMCs with TGF-β increases calcification, as well as increased VSMC migration and foam cell generation.^111^ Moreover, TGF-β1 osteo-inductive signalling involves the Smad2/3 pathway^112^ and SOX9-mediated^113^ up-regulation of RUNX2 in VSMCs. The up-regulation of osteoblast markers, such as Runx2, in VSMCs can be induced through incubation with many M1 inflammatory stimuli, including TNF-α, IL-6, and IL-18.^114–116^ Similarly, products of oxidative stress such as reactive oxygen species, a hallmark of the M1 macrophage phenotype, can also induce VSMC phenotypic switching to pro-calcifying.^117^ In addition, M1 macrophages can directly secrete oncostatin M, contributing to the development of atherosclerotic calcification by inducing osteoblastic transdifferentiation of VSMCs through the JAK3-STAT3 pathway.^118^ An auto/paracrine mechanism of M1-released BMP-2^119^ may have implications in VSMCs calcification via BMP-2 receptor/Smad1/5 signalling axis. The activation of Runx2, along with its chondrogenic downstream targets, can also induce VSMC apoptosis, as positive feedback for calcification nucleation, and is linked mechanistically to the DNA damage response.^120^ Hence, several interlinked mechanisms exist, in which macrophage-led inflammation synergistically intensifies active intimal vascular calcification.

## 4. Observed macrophage phenotype in macrocalcified plaque areas

In advanced calcified atherosclerotic plaques, macrophages surrounding areas of macrocalcification generally have acquired markedly less inflamed, more reparative phenotypes.^33^ The transition from inflamed micro- to stable macrocalcification is highly understudied. Macrocalcified plaque environments have not yet been successfully modelled in culture. Also, little research has been documented on how microcalcification in the arteries can transition into nodular or sheet-like structures, and whether this is influenced by the inflammatory state of the plaque, or if the structures themselves are influential factors. Certainly, differing macrocalcification structures contribute varyingly to the overall risk of rupture.^4^ CD68^+^ Mannose Receptor^+^ (M2-like) macrophages were stated to co-localize with cell-rich stable plaque areas, particularly away from the lipid core and rupture-prone shoulder regions of the plaque where M1-like cells tend to dominate.^69,121^ Furthermore, macrophages near macrocalcified deposits showed an M2-like phenotype.^122,123^ As stated, *in vitro* M2 macrophages are engaged in the healing response^103^ to plaque inflammation; and through the induction of VSMC osteoblastic differentiation,^19^ mediated mainly, but not exclusively by TGF-β signalling^124^ may be facilitating the macrocalcification process.^19^ But moreover, they can also reflect a level of calcification inhibiting activity.^37^

Overall, macrophage co-localization with calcification is reduced with larger calcifications and higher in microcalcified areas.^27^ Macrocalcified plaque appears to be enriched in tartrate resistant acid phosphatase (TRAP)-positive multinucleated giant cells and CD68^+^/Carbonic Anhydrase II/TRAP-positive osteoclast-like macrophages.^6,125^ These CD68^+^ MR^+^, CAII^+^, Cathepsin K (CATK)^low^ macrophages had minimal resorptive activity, indicating that RANK-L-led osteoclast-like changes within the plaque may not produce efficient osteoclasts from macrophages.^7,122^ Bone marrow-derived osteoclast-like cells could reduce calcified elastin mineral content *in vitro* by 80% whereas *in vivo*, osteoclasts induced elastin demineralization by 50%, without altering elastin integrity.^126^ This lack of efficiency may be caused by vascular cell production of soluble factors such as OPG and IL-18—shown to inhibit normal osteoclast generation and resorptive capability.^127^ Recent reports have shown that macrophage multinucleated giant cell formation in chronic inflammatory disease and osteoclast fusion in bone mass regulation display a common molecular signature.^128^ Research into the plaque proteome showed cartilage oligomeric matrix protein, a musculoskeletal and cardiovascular non-collagenous glycoprotein, can regulate macrophage phenotype within the atherosclerotic plaque and skew towards an alternatively activated and osteoclast-like phenotype.^129^

Macrophage–osteoclast interrelationship has been described in bone formation; a distinct population of osteolineage-associated resident macrophages, termed ‘osteal macrophages’ or osteomacs, has been recently described in mice; classified as F4/80^+^, TRAPc^−.130^ Interestingly, it was reported that osteomacs are injury-associated macrophage cells. They are present in high numbers in areas of bone matrix deposition during fracture healing processes. Their depletion suppressed bone healing *in vivo*^131,132^ and they were able to differentiate to multinucleated TRAP^+^ osteoclasts capable of bone resorption.^133^ Of note, true vascular ossification, with the presence of an established bone marrow-like region is greatly influenced by the anatomical location of the plaque and its originating arterial bed.^134,135^ For example, plaques in femoral arteries show a higher propensity for osteoid metaplasia than carotid plaques. Interestingly, a distinct myeloid origin circulating cell fraction expressing osteocalcin and bone alkaline phosphatase has pro-calcific activity *in vitro and in vivo*, contributing to ectopic vascular calcification in type 2 diabetes.^136^ While osteoclast-like cells in plaque may provide an interesting therapeutic potential due to their mineral removal and remodelling capability, it is yet unclear if exacerbating their presentation would be of benefit, since extreme enrichment of cathepsin K in atherosclerotic plaques could lead to redundant proteolytic plaque remodelling and plaque rupture.^137,138^ In line, calcified nodule formation is initiated in the regions of elastin degradation, therefore, a balance between osteo-immune cells is critical, yet subverted by aberrant and/or unresolved immune responses in atherosclerosis.^139^

## 5. Macrophage-osteoclast phenotypic switch

As mentioned above, lipids are important factors in the biomineralization process^140–142^; histologically, early calcification can be detected in acellular lipid pools in bone and ectopic mineralization.^143^ The lipid-rich necrotic core is the highest risk area to precursor micro- and macrocalcification deposits, and undergo long-term transformation into dense calcium phosphate.^65,66,144^ This notion is enhanced from studies showing that lesions with a higher load of calcification contain less lipid core.^145^ High serum LDL-cholesterol is highly correlated to vascular calcification,^146,147^ and both serum LDL and total cholesterol have been independently associated with CAC incidence.^148^ However, it remains elusive whether lipids are causative in atherosclerotic calcification or just represent an epiphenomenon, although they have been linked to calcification-associated phenotypes in macrophages.

Lipid handling equivalent processes can be drawn between macrophages and observed osteoclast-like traits. Although it is vastly under-investigated, this parallel may help to better understand the origin and role of osteoclast-like cells in the plaque. More specifically, foam cells, expressing the lysosomal protease CATK, have been shown to contribute to plaque remodelling,^149^ much like activated macrophages and osteoclast-like cells.^150^ Plaque multinucleated giant cells express markers such as TRAP and CATK along with their distinctive osteoclast morphological overlap.^6^ In culture, lipids and modified lipids have been shown to promote osteoclastogenesis through VSMC RANK-L up-regulation, direct macrophage osteoclast gene up-regulation, and promoting osteoclast survival.^151,152^ Similarly, lipid exposure in murine bone marrow-derived macrophages could trigger multinucleated giant cell formation in culture, a phenotype that could be greatly exacerbated by myeloid Mcl-1 depletion in Ldlr^−/−^ mice where a lipid accumulating, giant cell forming and apoptosis prone phenotype in macrophages was demonstrated.^153^ Furthermore, hyperlipidaemia in Ldlr^−/−^ mice, which is associated with increased plasma oxLDL levels, was seen to increase osteoclastogenesis potential in pre-osteoclasts *ex vivo.*^154^ Cochain *et al.*^48^ reported a triggering receptor expressed on myeloid cells 2 (TREM2) high, OPN expressing macrophage subset, probably foam cells, in single-cell sequencing of CD45^+^ cells isolated from the atherosclerotic aorta of Ldlr^−/−^ mice fed a western-type diet. This subset showed gene enrichment for lipid handling as well as osteoclast function and osteoclastogenesis. These cells were confirmed in meta-analysis with single-cell data from Kim *et al.* to be indistinguishable from foam cells and possess low inflammatory gene expression.^52,155^

As with the lipid crossover between ectopic calcification and bone, developing intimal calcification acquires the RANK/RANK-L/OPG axis, greatly influencing intimal calcification development and macrophage phenotypic switch. RANK-L drives osteoclastogenesis.^156^ VSMCs, stimulated by conditioned medium from inflammatory macrophages, up-regulate Runx2-controlled RANK-L production and secretion.^8^ Next to supporting osteoclast differentiation, RANK-L has been shown to induce IL-6 and TNF-α secretion in macrophages, and thus can reinforce the VSMC pro-calcifying phenotype.^157^ Partial deletion of Runx2 in VSMC in ApoE^-/-^ mice causing an alternative functional truncated Runx2 protein, showed reduced vascular calcification, with RANK-L expression reduction, reduced macrophage infiltration to the lesion, and reduced macrophage to osteoclast-like phenotypic switch.^158^ Further study with a VSMC-specific *runx2* deletion model showed reduced calcification, but no change in lipid metabolism, lesion size, or macrophage recruitment.^159^ This highlights a mechanism of vascular calcification possibly separable from the inflammatory and lipid-driven mechanisms.

It is possible that the osteoid metaplasia, driven by the pathways here highlighted, acts as a compensatory mechanism to control calcification progression, and moreover, as a mechanism of inflammation control and wound healing. The observations linking lipid handling to osteoclastogenesis may help to bridge the gap between highly inflammatory microcalcification-associated macrophages, and the osteoclastogenic switch capable of taking place. Understanding this association, and how it affects calcification development and progression, may be critical in future efforts to clinically modulate plaque inflammation and calcification.

## 6. Conclusions and future perspectives

Whilst calcification is an independent predictor of clinical cardiovascular events, the overall risk to plaque rupture or stability critically depends on the actual calcification phenotype. Bone-like vascular calcification has been shown to be a typical feature of more stable plaques and asymptomatic disease.^160,161^ However, even fibrocalcific plaques have an associated risk of adverse events, such as rupture, occlusion, or thrombosis through calcified nodules.^4^ Plaque regression studies and meta-analysis showed that a common feature of a regressing plaque is an increase in dense calcium volume and CAC score, which is inversely correlated to event risk.^22,162^ Treatment and rupture prevention, largely relies on aggressive lipid-lowering statin therapy, shown to stabilize plaques but also increase calcification. However, combination therapy with protein convertase subtilisin/kexin type 9 (PCSK9) could inhibit statin-induced calcification progression in 16 subjects, compared to statin monotherapy (*n* = 15) in a paired longitudinal study.^163^

Drugs to specifically treat or reverse atherosclerotic calcification are still currently missing. However, treating atherosclerotic calcification effectively at later stages will likely also not rely on purely targeting calcification, but through a better understanding of the greatest risks at each stage of the disease, so that treatment can be more targeted. Reducing inflammation and improving beneficial macrophage functions could represent a more powerful tailored strategy to prevent and reduce microcalcification, particularly in atherosclerosis patients with a high degree of vascular calcification, unresponsive to regular lipid-lowering therapy.

**Figure 1:**
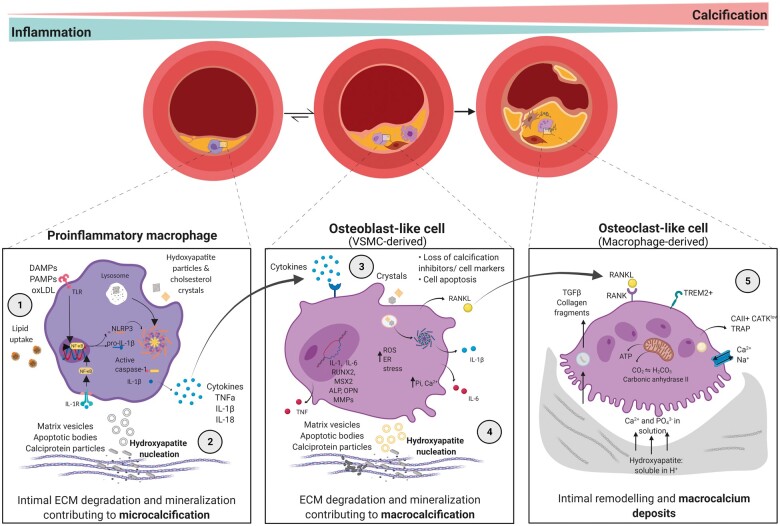
Interplay between macrophages and calcification in atherosclerotic plaque from early to late disease stage. Pro-inflammatory macrophages in the atherosclerotic plaque milieu undergo hydroxyapatite crystal and modified lipid uptake (1), as well as extracellular matrix vesicle and cytokine release (2). This establishes microcalcification in the plaque and induces an osteoblast-like phenotype in VSMCs (3). A pro-calcifying microenvironment and the osteochondrogenic switch of VSMCs, driven by macrophages, increase inflammatory calcification deposition and establish more densely calcified nodules (macrocalcification) (4). Macrophage engagement in the receptor activator of nuclear factor kappa B (RANK)/RANK-Ligand axis results in alternatively activated, remodelling-associated, and osteoclast-like macrophages surrounding macrocalcification deposits (5). As such, the inflammatory burden in this plaque microenvironment falls, with increased calcification and remodelling. ALP, alkaline phosphatase; ATP, adenosine triphosphate; Ca2^+^, calcium; CAII, carbonic anhydrase 2; CATK, cathepsin K; DAMPs, damage-associated molecular patterns; ECM, extracellular matrix; ER, endoplasmic reticulum; IL-1R, interleukin-1 receptor; IL-1β, interleukin 1 beta; IL-6, interleukin 6; IL-18: interleukin 18; MMPs, matrix metalloproteinases; MSX2, Msh Homeobox 2; NLRP3, NLR family pyrin domain containing 3; OPN, osteopontin; PAMPs, pathogen-associated molecular patterns; Pi, phosphate; ROS, reactive oxygen species; RUNX2, runt-related transcription factor 2; SASP, senescence-associated secretory phenotype; TGFβ, transforming growth factor-beta; TLRs, toll-like receptors; TNF-α, tumour necrosis factor-alpha; TRAP, tartrate resistant acid phosphatase; TREM2, triggering receptor expressed on myeloid cells 2.

A research explosion has occurred based on the role of macrophages in the process of vascular calcification and is summarized in *Figure 1*. The phenotypic plasticity and functional heterogeneity of macrophages according to the microenvironment variables led to the understanding of their pleiotropic effects in the atherosclerotic plaque calcification. Inflammatory macrophage activity accelerates plaque calcification profoundly, through many mechanisms that also couple to plaque growth and risk of rupture. Meanwhile in-kind, a calcified microenvironment reinforces these processes and produces calcification-associated macrophage phenotypes linked to macrocalcification. As such, net M2 skewing in atherosclerosis through clinical intervention may reduce not only plaque progression but also calcification growth, both in early and late stages. Reducing microcalcification generation, and enhancing fibrotic activity associated with established stable calcification; specifically through inflammation reduction, direct calcification inhibition activity and regulation of plaque cell death. The precise modulation mechanisms that allow for *in vivo* differentiation of macrophages into a phenotype in a manner that is more protective for the patient is still an unmet need and an urgent problem to be solved. Therefore, deep phenotyping of macrophages subsets with high-resolution omic methodologies like single-cell technology in calcified plaques is still an open research area and represents a clear benefit for better disease understanding and assessment of clinical risk. However, the relative contribution of macrophages to late-stage calcification and disease state is also yet to be comprehensively elucidated. Comparative assessment of cellular phenotypes presenting at all stages of vascular calcification can help to fill in many gaps in understanding of how this contributes to disease progression, risk of plaque rupture (i.e. clinical events) and how current therapeutic strategies may be improved. Conclusively, additional investigation of the potential molecular mechanism and function of how macrophages modulate the progression and regression of vascular calcification is expected not only to bridge the gap between *in vitro* and *in vivo* observations but also to uncover a new notion for the prevention and treatment of vascular calcification.

## Data Availability

This review article does not contain new original data.
